# Executive and Social Functioning in Children and Adolescents With Noonan Syndromes: Cognition and Behavior

**DOI:** 10.1016/j.jaacop.2025.05.002

**Published:** 2025-05-21

**Authors:** Jennifer Kramer, Renée L. Roelofs, Ellen Wingbermühle, Sara Pieters, Jos Egger

**Affiliations:** a Donders Institute for Brain, Cognition and Behaviour, Radboud University, Nijmegen, the Netherlands; b Center of Excellence for Neuropsychiatry, Vincent van Gogh Institute for Psychiatry, Venray, the Netherlands; c Department of Human Genetics, Radboud University Medical Center, Nijmegen, the Netherlands; d Behavioural Science Institute, Radboud University, Nijmegen, the Netherlands; e Karakter, Child and Adolescent Psychiatry, Nijmegen, the Netherlands; f Stevig, Specialized and Forensic Care for People with Intellectual Disabilities, Dichterbij, Oostrum, the Netherlands

**Keywords:** Noonan syndromes, RASopathies, executive functions, social cognition, contextual neuropsychology

## Abstract

**Objective:**

The current study aims to examine executive and social functioning in children and adolescents with Noonan syndromes, which contributes to the understanding of the cognitive and behavioral profile of this population and possible treatment options.

**Method:**

A total of 26 children and adolescents with Noonan syndromes (including Noonan syndrome, Noonan syndrome with multiple lentigines, and Noonan-like syndrome with loose anagen hair; mean age = 11.92 years, SD = 2.64) and 25 typically developing children and adolescents (mean age = 10.32 years, SD = 2.75) participated in this study. Cognitive and behavioral measures of executive and social functioning of children and adolescents in these groups were compared using multivariate analyses of variance. Moreover, the relationship between executive and social functioning was examined.

**Results:**

Results showed significant group differences on working memory and attention, with controls outperforming children and adolescents with Noonan syndromes, even when controlling for crystallized intelligence. At a behavioral level, children and adolescents with Noonan syndromes experienced more executive function problems and more characteristics of attention-deficit/hyperactivity disorder and autism spectrum disorders in daily life than controls, even when controlling for crystallized intelligence. Positive relationships were found between behavioral measures of executive functions and characteristics of autism spectrum disorders.

**Conclusion:**

Difficulties in working memory and attention seem to be key cognitive features in children and adolescents with Noonan syndromes. These difficulties occur alongside parental reports of executive function problems, characteristics of attention-deficit/hyperactivity disorder, and autism spectrum disorders.

## Plain language summary

This Netherlands study examined executive and social functioning in 26 children and adolescents (7-17 years) with Noonan syndromes compared to 25 typically developing peers. Youth with Noonan syndromes showed relatively lower performance on working memory and attention, and in daily functioning. Parents reported more executive function problems, characteristics of attention deficit with hyperactivity, and autism spectrum disorders in the group with Noonan syndromes. These findings contribute new knowledge of cognitive and behavioral features of Noonan syndromes and underline the importance of individualized neuropsychological assessment in this population, especially in the context of functional impairments, such as learning and social problems.

Noonan syndrome (NS) and related disorders, such as Noonan syndrome with multiple lentigines (NSML), Noonan-like syndrome with loose anagen hair (NSLH), Noonan syndrome-like disorder (CBL), cardiofaciocutaneous syndrome (CFCS), and Costello syndrome (CS), are a group of multisystem disorders that are caused by genetic variants affecting the Ras-MAPK signaling pathway and are therefore also called RASopathies together with neurofibromatosis type 1 (NF1) and Legius syndrome.^1^ Noonan syndrome and related disorders share clinical characteristics, such as distinct facial features, short stature, congenital heart defects, developmental delay, and neurological difficulties, although individual variation is large.^1^ Moreover, these syndromes are frequently accompanied by cognitive difficulties and behavioral problems, which vary greatly in frequency and severity among individuals.^2^ NS occur in approximately 1 in every 1,000 to 2,000 live births.^1^ In this article, we focus on NS, NSML and NSLH, which we here refer to as “Noonan syndromes.” To date, more than 19 different genes have been identified that are associated with Noonan syndromes. The most common are *PTPN11*, *SOS1, SOS2, RAF1, RIT1, KRAS, HRAS*, and *LZTR1*.^3^ Variations in *PTPN11* are most commonly involved in NS and NSML, whereas variations in *RAF1* are also mainly involved in NSML. NSLH is mostly caused by variations in *SHOC2* and *PPP1CB*.

Children and adolescents with Noonan syndromes show lower intellectual abilities than children and adolescents without Noonan syndromes, although the range is wide and mean IQ generally lies in the (low) average range (Full Scale Intelligence Quotient: 85-95).^4^ Moreover, children with Noonan syndromes are more likely to show delayed motor and language development. Besides relatively lower intellectual abilities and delayed motor and language development, cognitive difficulties have been described in many different domains. It appears that difficulties in executive functions and social cognition are most common.^2^^,^^5^

Executive functions refer to higher-order cognitive processes required to organize information and to control behavior. Executive functions are especially needed in complex, new, and ambiguous situations, when automatic behavior is insufficient to achieve set goals.^6^^,^^7^ Miyake *et al.*^8^ identified 3 core cognitive processes that enable individuals to regulate and adapt their behavior. These processes include inhibitory control, working memory, and cognitive flexibility. Other researchers identified 9 executive functions, including working memory, inhibitory control, cognitive flexibility, attention, planning, organisation, self-monitoring, initiation, and emotion regulation.^9^ These executive function processes interact with each other as an overarching system to perform complex behavior.^10^ Attention is related to the selective process that enables efficient information processing by focusing only on relevant information and ignoring irrelevant information. Selecting relevant information over irrelevant information in the flood of information requires cognitive control, which is carried out by executive functions.^11^ Problems in selective attention involve less ability to focus purposefully on specific aspects of the environment and more difficulties ignoring stimuli or inhibiting responses. Sustained attention includes actively focusing or distributing attention over longer periods of time.^11^ Social cognition refers to mental processes that are required for social interaction and are necessary for recognizing, interpreting, and responding to social information.^12^ It includes, among others, emotion recognition (ie, the decoding of social information, for example by facial expression) and Theory of Mind (ie, the interpretation of social information in terms of mental states and dispositions).^13^

With regard to executive functions, studies have shown that children and adolescents with NS have significantly more impairments in inhibitory control, sustained attention, and auditory attention compared to their unaffected siblings.^14^ In particular, the ability to regulate attention seems to be impaired in children with NS.^15^ Another study also found significant differences between children with and without NS on an auditory attention task and an inhibition task, although these differences were not significant after controlling for intelligence.^16^ In terms of working memory, Pierpont *et al.*^14^ found weaker performance on an auditory and visual working memory task in children with NS when compared to normative data. Of these children, 34% even showed significant impairment. At the behavioral level, parents reported that their children with NS experienced more frequent working memory problems, attention problems, hyperactivity, and general executive function problems in daily life than their unaffected children.^5^^,^^14^^,^^16^ Moreover, children and adolescents with NS are more likely to meet the criteria for attention-deficit/hyperactivity disorder (ADHD) than children and adolescents without NS.^14^ Group differences at the behavioral level remained significant after controlling for intelligence in children with NS.^16^

Although structural and functional imaging studies in children and adolescents with Noonan syndromes are limited, there are indications for hyperconnectivity in the visual, ventral attention, left frontoparietal, and limbic networks in children with NS compared with a control group, which may be related to impairments in inhibition, attention, and orientation abilities.^17^ Another study on brain structures in children with NS found that the size of the caudate, putamen, and pallidum of the corpus striatum was smaller compared with those of typically developing children. These brain structures are, among others, associated with motivation, motor and action planning, decision making, and reinforcement. A reduced size of the corpus striatum is often associated with inattentiveness and hyperactivity, symptoms that are frequently observed in children with NS.^18^

Concerning social cognition, there is only one study that used cognitive neuropsychological tasks in children with NS. This study showed significantly lower performance on emotion recognition and Theory of Mind in children with NS compared to children without NS, although the differences did not remain significant after controlling for intelligence.^16^ However, social–emotional development is frequently delayed in children and adolescents with NS, and—at the behavioral level—problems in understanding social situations, articulating and explaining social experiences, and problems in connecting with peers are frequently mentioned.^19^^,^^20^ Parents perceive that their children with NS have fewer social skills than their unaffected children.^5^^,^^14^ Emotional dysregulation is also frequently reported in children and adolescents with NS.^21^ Moreover, there is an increased likelihood of characteristics of autism spectrum disorders (ASD).^22^^,^^23^

To date, only a few controlled studies with relatively small sample sizes have examined executive and social functioning in children and adolescents with Noonan syndromes. The main aim of the current study is to examine cognitive and behavioral measures of executive and social functioning in children and adolescents with Noonan syndromes, compared to a typically developing control group. This contributes to a better understanding of the cognitive and behavioral profile of children and adolescents with Noonan syndromes and, as a result, to possible treatment options. First, the performance of children and adolescents with and without Noonan syndromes on cognitive measures of executive and social–cognitive functioning (ie, working memory, inhibitory control, cognitive flexibility, sustained attention, emotion recognition, and Theory of Mind) is compared (with and without controlling for crystallized intelligence). Second, behavioral measures of executive functioning, ADHD characteristics, and ASD characteristics are compared between the 2 groups, with and without controlling for crystallized intelligence. Although Full Scale Intelligence Quotient generally lies in the (low) average range (85-95) in children with Noonan syndromes, hardly any previous study of executive and social–cognitive functioning in children with Noonan syndromes controlled for intelligence. Therefore, cognitive impairments may have been overestimated in these studies.^24^ The only studies that controlled for intelligence found no specific cognitive impairments in patients with Noonan syndromes.^2^^,^^16^ However, these studies controlled for Full Scale Intelligence Quotient, which summarizes abilities in the areas of fluid reasoning, visual–spatial, working memory, and processing speed, in addition to verbal comprehension.^25^ Especially fluid reasoning, which is the ability to identify and apply rules through logical reasoning, such as recognizing the underlying conceptual relationship between visual objects, is strongly related to executive functioning.^26^ It is therefore possible that an overcorrection for weaknesses in executive functioning occurred, resulting in a lack of significant group differences. Therefore, our study examines group differences both with and without controlling for crystallized intelligence to provide a complete picture of the cognitive issues in this population. Crystallized intelligence is defined as a person’s acquired language-based knowledge against the background of her or his culture and environment^27^ and is therefore a good representation of general intellectual capacities without a strong reliance on executive functioning.

We expect significantly weaker cognitive performances on executive function and social cognition in children and adolescents with Noonan syndromes compared to controls, also after controlling for crystallized intelligence. Regarding behavioral measures, we expect more executive function problems to be experienced in daily life, and more characteristics of ADHD and ASD in the Noonan syndromes group, regardless of controlling or not controlling for crystallized intelligence.

Finally, the current study examines to what extent cognitive and behavioral measures of executive functioning are related to cognitive and behavioral measures of social functioning in children and adolescents. We expect to find a positive relationship between executive and social (cognitive) functioning in children and adolescents with and without Noonan syndromes, because this relation was also found in children with ADHD, ASD, and focal epilepsy and typically developing children.^28^, ^29^, ^30^

## Method

### Participants

For the current study, 51 children and adolescents were recruited: 26 children and adolescents with Noonan syndromes and 25 children and adolescents without Noonan syndromes. Most children and adolescents in the patient group had been diagnosed with NS (92.3%). One child was diagnosed with NSML (3.9%) and one with NSLH (3.9%). The underlying pathogenic variants included *PTPN11* (34.6%), *KRAS* (23.1%), *SOS1* (19.2%), *LZTR1* (7.7%), and *PPP1CB* (3.9%). For 3 children and adolescents (11.5%), no information was available on genetic testing or on the pathogenic variant; the diagnosis was based on clinical criteria only. The participants in the control group were typically developing children and adolescents. The age of children and adolescents with Noonan syndromes ranged from 7 to 17 years (mean = 11.92 years, SD = 2.64), and 30.8% were boys. The age for children and adolescents without Noonan syndromes ranged from 7 to 16 years (mean = 10.32 years, SD = 2.75), and 40.0% were boys. There was no significant difference in gender distribution (χ^2^[1] = 0.48, *p* = .565) between the groups. There were significant differences between the groups in age (*t*[49] = −2.125, *p* = .039, *d* = −0.595) and Verbal Comprehension Index (Noonan syndromes: mean = 94.50, SD = 13.76, range = 73-127; controls: mean = 114.04, SD = 12.66, range = 95-134; *t*[49] = 5.271, *p* < .001, *d* = 1.477) as measured with the Wechsler Intelligence Scale for Children, Fifth Edition (WISC-V-NL).^25^

### Measures

The Dutch versions of standardized and widely used neuropsychological tests were administered to assess crystallized intelligence (ie, Verbal Comprehension Index of the WISC-V^25^), working memory (ie, Digit Span subtest of the WISC-V^25^), sustained attention (ie, Concentration performance of the D2 Test of Attention^31^), inhibitory control (ie, Color Word Interference Test [CWIT] of the Delis–Kaplan Executive Function System [D-KEFS]^32^), cognitive flexibility (ie, Trail Making Test [TMT] of the D-KEFS^32^), emotion recognition (ie, subtest Affect Recognition of the Developmental Neuropsychological Assessment [NEPSY-II]^33^), and Theory of Mind (ie, Theory of Mind subtask of the NEPSY-II^33^). Moreover, questionnaires were administered to assess everyday executive function problems experienced in the home environment of children and adolescents reported by parents (ie, Behavior Rating Inventory of Executive Function [BRIEF]^34^), ADHD characteristics reported by parents (ie, ADHD-questionnaire [AVL]^35^), and ASD characteristics reported by parents (ie, Autism spectrum questionnaire [ASV^36^]). A detailed description of the neuropsychological tests and questionnaires used is provided in Supplement 1, available online.

### Procedure

All procedures in the current study were conducted in accordance with the Declaration of Helsinki and approved by the Institutional Review Board of the Vincent van Gogh Institute for Psychiatry (20.02527, 21.12.2021) and by the Ethics Committee of the Faculty of Social Sciences (ECSW) of Radboud University (ECSW-2021-156, 17.03.2022). Participants with Noonan syndromes were recruited by the Centre of Excellence for Neuropsychiatry of the Vincent van Gogh Institute for Psychiatry in the Netherlands via the Dutch Noonan Syndrome Foundation (eg, Web page and social media of the Dutch Noonan Syndrome Foundation). For some children and adolescents with Noonan syndromes, the current study was part of a broader study on the effects of an executive function training. The baseline measurement of these children and adolescents were used for the current study. Children and adolescents without Noonan syndromes were also recruited via the Dutch Noonan Syndrome Foundation (ie, siblings of children and adolescents with Noonan syndromes) and the networks of the researchers, the students who assisted with data collection, and participating children and adolescents and their parents. A few children and adolescents with Noonan syndromes in the current study were also described in an earlier study on the cognitive phenotype and psychopathology.^2^

Inclusion criteria for this study were age between 7 and 17 years, sufficient command of the Dutch language, primary education in the Netherlands (currently attending or completed), no intellectual disability (intellectual disability defined as Verbal Comprehension Index <70), ability to complete neuropsychological tasks and questionnaires, and the presence of a parent or caregiver who was able to complete questionnaires about the child’s or adolescent’s behavior. In addition, for children and adolescents with Noonan syndromes, a confirmed genetic diagnosis by a clinical geneticist (clinical or confirmed by genetic testing) was required. Children and adolescents with a previously diagnosed psychiatric disorder were excluded from the control group.

Participation occurred on a voluntary basis. Children, adolescents, and their parents received an information letter prior to their participation. The data collection was performed during house visits or at locations of the Centre of Excellence for Neuropsychiatry of the Vincent van Gogh Institute for Psychiatry. First, participants and their parents were informed about the test procedure and had the opportunity to ask questions about the information letter. If children and adolescents and their parents agreed to participate, a declaration of consent was signed by both the children and adolescents and their parents. Parents were asked to complete questionnaires about the behavior of their children and adolescents while the neuropsychological examination was being conducted. Neuropsychological assessment of the child or adolescent took approximately 3 hours and was conducted by trained psychologists. After the assessment, participants received a small thank-you present (ie, a voucher of €10).

### Data Analysis

Data were analyzed using IBM SPSS Statistics version 29.0. First, missing data were investigated. One child did not complete the D2 test of attention. For 7 children, no questionnaires were completed by their parents. The results of those children were not included in the respective analyses. With regard to missing data in the questionnaires completed by parents, the following procedure was applied: if an item of a subscale was missing, the item was replaced by the mean of the whole subscale. In total and across all participants, one item was not completed for the ASV, and 10 items were not completed for the BRIEF. In 1 participant’s questionnaire, 2 items of a subscale were missing; in all other subjects, at most 1 item of a subscale was missing.

The assumptions of normality, homogeneity, and linearity were checked before each analysis. The assumptions were not violated, and the planned analyses could be conducted.

First, independent-samples *t* tests were performed to examine group differences with respect to age and crystallized intelligence (ie, Verbal Comprehension Index). To explore group differences with regard to gender distribution, a χ^2^ test was performed.

Next, 4 multivariate analyses of covariance (MANCOVA) were conducted: (1) MANCOVA with group as between-subject factor, age as covariate and working memory, inhibitory control, cognitive flexibility, sustained attention, emotion recognition, and Theory of Mind as dependent variables; (2) MANCOVA with group as between-subject factor, age, and crystallized intelligence as covariates and working memory, inhibitory control, cognitive flexibility, sustained attention, emotion recognition, and Theory of Mind as dependent variables; (3) MANCOVA with group as between-subject factor, age as covariate and the behavioral measures executive function problems experienced in daily life, ADHD characteristics, and ASD characteristics as dependent variables; and (4) MANCOVA with group as between-subject factor, age, and crystallized intelligence as covariates and the behavioral measures executive function problems experienced in daily life, ADHD characteristics, and ASD characteristics as dependent variables.

Finally, 3 multiple regression analyses were conducted to examine the relationship between cognitive and behavioral measures of executive and social functioning in children and adolescents. In the first regression analysis, emotion recognition was the dependent variable and Noonan syndromes (yes/no), age, crystallized intelligence, and the executive function measures (ie, working memory, inhibitory control, cognitive flexibility, sustained attention, and executive function problems experienced in daily life as reported by parents) were independent variables. In the second and third regression analyses, the dependent variables were Theory of Mind and ASD characteristics, respectively. The independent variables were the same as those in the first regression analysis. *Z* scores were used for the quantitative independent variables in all 3 regression analyses.

## Results

### Multivariate Analyses of Covariance

Results of the first MANCOVA showed significant differences between children and adolescents with and without Noonan syndromes on cognitive measures when controlling for age (*F*_6,42_ = 4.519, *p* = .001, Wilks’ *Λ* = 0.608, partial *η*^*2*^ = 0.392). More specifically, significant group effects were found on sustained attention, working memory, and emotion recognition after controlling for age, with medium to large effect sizes. Controls performed better on these cognitive domains than children and adolescents with Noonan syndromes. No other significant group differences were found. Results are shown in Table 1.

**Table 1:** Multivariate Analyses of Covariance on (Social) Cognitive Measures

	Controls (n = 25)	Children and adolescents with Noonan syndromes (n = 25)	Univariate test with age as covariate		Effect size with age as covariate	Univariate test with age and crystallized intelligence as covariates		Effect size with age and crystallized intelligence as covariates
	Mean (SD)	Mean (SD)	*F*_1,47_	*p*	Partial *η*^*2*^	*F*_1,46_	*p*	Partial *η*^*2*^
Working memory	27.20 (5.49)	23.96 (5.91)	12.383	<.001	0.209	4.282	.044	0.085
Inhibitory control	1.86 (0.36)	2.04 (0.60)	2.876	.097	0.058	0.163	.689	0.004
Cognitive flexibility	4.59 (1.21)	4.65 (1.21)	0.116	.735	0.002	0.219	.642	0.005
Sustained attention	136.12 (38.72)	120.28 (34.54)	22.228	<.001	0.321	11.809	.001	0.204
Emotion recognition	27.00 (3.16)	24.56 (4.94)	6.706	.013	0.125	1.008	.321	0.021
Theory of Mind	23.24 (3.27)	23.32 (2.46)	0.641	.427	0.013	2.126	.152	0.044

Note: Controls = children and adolescents without Noonan syndromes.

Results of the second MANCOVA showed significant differences between children and adolescents with and without Noonan syndromes on cognitive measures when controlling for age and crystallized intelligence (*F*_6,41_ = 2.608, *p* = .031, Wilks’ *Λ* = 0.724, partial *η*^*2*^ = 0.276). More specifically, significant differences between groups were found on working memory and sustained attention, with medium and large effect sizes. Controls performed better on these cognitive domains than children and adolescents with Noonan syndromes. The difference between the groups on emotion recognition did not remain significant when controlling for age and crystallized intelligence. Results are shown in Table 1.

Results of the third and fourth MANCOVA showed significant differences between children and adolescents with and without Noonan syndromes on all behavioral measures when controlling for age (*F*_3,39_ = 24.410, *p* < .001, Wilks’ *Λ* = 0.347, partial *η*^*2*^ = 0.653) and when controlling for age and crystallized intelligence (*F*_3,38_) = 16.359, *p* < .001, Wilks’ *Λ* = 0.436, partial *η*^*2*^ = 0.564). More specifically, significant differences between groups were found in executive function problems experienced in daily life, ADHD characteristics, and ASD characteristics, with more executive function problems experienced in daily life, ADHD, and ASD characteristics in the group with Noonan syndromes (large effect sizes). Results are shown in Table 2.

**Table 2:** Multivariate Analyses of Covariance on Behavioral Measures

	Controls (n = 24)	Children and adolescents with Noonan syndromes (n = 20)	Univariate test with age as covariate		Effect size with age as covariate	Univariate test with age and crystallized intelligence as covariates		Effect size with age and crystallized intelligence as covariates
	Mean (SD)	Mean (SD)	*F*_1,41_	*P*	Partial *η*^*2*^	*F*_1,40_	*p*	Partial *η*^*2*^
Experienced EF problems	97.83 (20.51)	156.55 (25.37)	67.459	<.001	0.622	41.649	<.001	0.510
ADHD characteristic	9.46 (8.55)	36.05 (11.92)	72.409	<.001	0.638	50.479	<.001	0.558
ASD characteristic	38.33 (11.53)	64.20 (15.57)	37.225	<.001	0.476	24.126	<.001	0.376

Note: ADHD = attention-deficit/hyperactivity disorder; ASD = autism spectrum disorder; Controls = children and adolescents without Noonan syndromes; EF = executive function.

### Multiple Regression Analyses

Prior to the regression analyses, bivariate correlations were calculated (Table S1, available online). The model of the first regression analysis, with emotion recognition as dependent variable and Noonan syndromes (yes/no), the *Z* scores of age, crystallized intelligence, and executive function measures (ie, working memory, inhibitory control, cognitive flexibility, sustained attention, and executive function problems experienced in daily life as rated by parents) as independent variables was not significant (*F*_8,35_ = 1.405, *p* = .229, adjusted *R*^*2*^ = 0.070). The levels of the independent variables did not predict the levels of emotion recognition. Results are shown in Model 1 in Table 3.

**Table 3:** Multiple Regression Analyses: Model 1 (Emotion Recognition as Dependent Variable), Model 2 (Theory of Mind as Dependent Variable), Model 3 (ASD Characteristics as Dependent Variable)

	*β*	Model 1				*β*	Model 2				*β*	Model 3			
		*b*	SE *B*	*t*	*p*		*b*	SE *B*	*t*	*p*		*b*	SE *B*	*t*	*p*
Noonan syndromes	–0.180	–1.445	2.347	–0.616	.542	.277	1.590	1.380	1.152	.257	.043	1.611	5.813	0.277	.783
Age	.203	0.809	1.004	0.806	.426	.330	0.939	0.591	1.589	.121	.077	1.418	2.487	0.570	.572
Crystallized intelligence	.132	0.563	0.812	0.694	.492	.367	1.121	0.478	2.346	.025	.060	1.181	2.012	0.587	.561
Sustained attention	.287	1.105	1.104	1.001	.324	.205	0.565	0.649	0.870	.390	.014	0.253	2.734	0.092	.927
Working memory	–0.202	–0.785	0.824	–0.952	.348	–0.132	–0.367	0.485	–0.758	.454	–0.075	–1.347	2.042	–0.660	.514
Inhibitory control	.062	0.349	1.060	0.329	.744	–0.039	–0.157	0.624	–0.251	.803	.151	3.930	2.626	1.497	.143
Cognitive flexibility	–0.138	–0.543	0.699	–0.776	.443	–0.274	–0.773	0.411	–1.879	.069	–0.109	–1.978	1.732	–1.142	.261
EF problems	–0.108	–0.440	1.114	–0.395	.695	–0.031	–0.090	0.655	–0.138	.891	.786	14.762	2.759	5.350	<.001

Note: Standardized values of the quantitative independent variables were used. ASD = autism spectrum disorder; EF = executive function.

The model of the second regression analysis, with Theory of Mind as dependent variable and Noonan syndromes (yes/no), the *Z* scores of age, crystallized intelligence, and executive function measures (ie, working memory, inhibitory control, cognitive flexibility, sustained attention, and executive function problems experienced in daily life as rated by parents) as independent variables was significant (*F*_8,35_ = 4.145, *p* = .001, adjusted *R*^*2*^ = 0.369). A total of 36.9% of the variance of Theory of Mind could be explained by the independent variables. Crystallized intelligence was positively related to Theory of Mind, indicating that higher levels of crystallized intelligence (represented by a higher score on the VBI) were related to stronger skills in Theory of Mind. No other significant relationships were found. Results are shown in Model 2 in Table 3.

The model of the third regression analysis, with ASD characteristics as dependent variable and Noonan syndromes (yes/no), the *Z* scores of age, crystallized intelligence, and executive function measures (ie, working memory, inhibitory control, cognitive flexibility, sustained attention, and executive function problems experienced in daily life as rated by parents) as independent variables was significant (*F*_8,35_ = 15.647, *p* < .001, adjusted *R*^*2*^ = 0.732). A total of 73.2% of the variance of ASD characteristics could be explained by the independent variables. Executive function problems were positively related to ASD characteristics, indicating that more parent-rated executive function problems in daily life were associated with more parent-rated ASD characteristics. No other significant relationships were found. Results are shown in Model 3 in Table 3.

## Discussion

The main aim of the study was to examine executive and social functioning in children and adolescents with Noonan syndromes compared to children and adolescents without Noonan syndromes, with and without controlling for crystallized intelligence. In addition, the relation between executive and social (cognitive) functioning was investigated.

The results regarding executive functioning showed a significantly higher performance on working memory and sustained attention in children and adolescents without Noonan syndromes, when controlling for age. The group differences remained significant when controlling for age and crystallized intelligence. Difficulties in working memory and sustained attention seem to be key cognitive features in children and adolescents with Noonan syndromes, which are not associated with lower intelligence levels. The results were partly in line with our expectations. Some previous studies showed that children and adolescents with Noonan syndromes had significantly more impairments in executive functioning. However, these studies did not control for intelligence. The only studies that controlled for overall intelligence and/or educational level found no specific cognitive impairments in patients with Noonan syndromes.^2^^,^^16^ One explanation for why we found a group difference on attention and working memory and the other 2 studies did not is that the other 2 studies controlled for Full Scale Intelligence Quotient, which also summarizes abilities in the area of fluid reasoning.^25^ Especially fluid reasoning is strongly related to executive functioning.^26^ Therefore, it is possible that an overcorrection for weaknesses in executive functioning took place, which resulted in a lack of significant group differences. By controlling only for crystallized intelligence (which does not heavily rely on executive functioning) in our study, we found a difference between children and adolescents with and without Noonan syndromes in working memory and attention. These basic executive functions are important for organizing information and controlling behavior, and—among other executive functions—are especially needed in complex, novel, and ambiguous situations, when automatic behavior is insufficient to achieve set goals.^10^ Difficulties in these key executive functions may explain the daily life problems in social functioning reported by parents. Contrary to our expectations, no group differences were found in inhibitory control and cognitive flexibility. Specific task characteristics may play a role here. Research suggests that the commonly used and well-described task that we used in this study for measuring inhibitory control may not be optimal to capture individual differences, because of a small amount of variation.^37^ The authors assume that this also applies to other neuropsychological tasks with a small amount of variation, such as the Trail Making Test for measuring cognitive flexibility.

The results regarding social–cognitive functioning showed a significantly higher performance on emotion recognition in children and adolescents without Noonan syndromes, when controlling only for age. When controlling for age and crystallized intelligence, children and adolescents with Noonan syndromes showed good emotion recognition and Theory of Mind equal to those of children and adolescents without Noonan syndromes. The results did not meet our expectations. However, the results confirm the previous results of the studies of Naylor *et al.*^16^ and Wingbermühle *et al.,*^2^ which describe social difficulties experienced in daily life in children and adults with Noonan syndromes in the absence of severe social impairments. At the behavioral level, children and adolescents with Noonan syndromes were found to experience more executive function problems in daily life and more ADHD and ASD characteristics as rated by parents compared to controls, when controlling for age and when controlling for age and crystallized intelligence. The results were consistent with our expectations and with previous studies in which more problems at the behavioral level were found in children and adolescents with Noonan syndromes compared with typically developing children.^5^^,^^16^ Interestingly, there seems to be a discrepancy between the results on cognitive and behavioral measures in children and adolescents with Noonan syndromes. On the one hand, neuropsychological tasks, particularly social–cognitive measures, may not seem to capture the problems in social–emotional functioning as seen by parents/caregivers and clinicians regarding children and adolescents with Noonan syndromes in daily life (among which are emotion regulation and a delay in social emotional development). Up to now, neuropsychological tasks of social cognition cannot fully reflect the complexity of social–cognitive processes in everyday life, consisting of recognizing, interpreting, and responding to social information.^38^ For example, recognizing emotions in daily life does not involve static pictures but, rather, moving facial expressions, which change rapidly and occur in different contexts. Emotion recognition in everyday life is therefore much more complex than what is captured in a neuropsychological task, and may thus rely more heavily on basic information processing such as working memory and attention. On the other hand, parental behavioral measures may also be biased, for example, by the parent’s own characteristics or by the parent’s expectations about the child’s overall abilities.^39^

The results regarding the relationship between executive and social (cognitive) functioning in children and adolescents were not completely in agreement with our expectations. A positive relationship between executive and social (cognitive) functioning was expected, because this was also found in children and adolescents with ADHD, ASD, and focal epilepsy and in typically developing children.^28^, ^29^, ^30^ However, in our study, this relationship was found only for the behavioral measures reported by parents, indicating that more executive function problems experienced in daily life were related to more ASD characteristics in children and adolescents. No relationships were found between cognitive measures of executive functioning and emotion recognition or Theory of Mind. Again, the lack of a relationship between executive function measures and social (cognitive) measures could be explained by the aforementioned and known limitations of cognitive measures of (social) cognitive functioning and the rather small sample size, especially for the multiple regression analyses. Moreover, no relationships were found between group (children and adolescents with Noonan syndromes and children and adolescents without Noonan syndromes) and emotion recognition, Theory of Mind, or ASD characteristics. Given the fact that multiple independent variables were included in the regression models, this can reflect a power problem, and the group effects should be interpreted with caution.

This study contributes to the body of evidence regarding executive and social functioning of children and adolescents with Noonan syndromes. Overall strengths of this study include the use of a comprehensive neuropsychological assessment, consisting of cognitive and behavioral measures of executive and social functioning, with and without controlling for crystallized intelligence, the relatively large sample of children and adolescents with Noonan syndromes, and the use of a control group consisting of unaffected siblings and other typically developing children and adolescents. Even though the sample size was quite small, especially for multiple regression analyses, the sample size can be considered a good reflection of the relatively low prevalence of children and adolescents with Noonan syndromes. Because the age range of the children and adolescents was relatively large, the results could not be specified to a specific age group. It is also important to note that almost all children and adolescents were diagnosed with NS, only one child with NSML, and one child with NSLH. The results are therefore not generalizable to other RASopathies. Because children and adolescents with intellectual disabilities were not included in the current study, the results cannot be generalized to individuals with an intellectual disability. However, intellectual disability occurs in a minority of children and adolescents with Noonan syndromes, and the mean (crystallized) intelligence score of the children and adolescents in the current study strongly aligns with the intelligence scores that are mentioned in other studies. Moreover, we used only reports from parents on the behavior of the children and adolescents and no other source of observational reports, for example, from teachers. Another limitation of the current study is that we did not control for medication use. Finally, in line with previous research, we used only explicit social–cognitive tasks, which highly depend on language and higher-order cognitive functions. Explicit processes within social interactions are controlled and conscious, whereas implicit processes are automatic and sometimes even unconscious.^40^ Explicit cognition and implicit cognition are thought to jointly predict behavior.^41^ For future studies, it would be interesting to use social–cognitive measures of implicit social cognition (ie, more automatic and impulsive processes of social behavior) to capture social–cognitive processes in all its facets in children and adolescents with Noonan syndromes.

Our findings indicate that children and adolescents with Noonan syndromes have poorer working memory and sustained attention, as well as more executive function problems in daily life, ADHD, and ASD characteristics (all parent-rated) than children and adolescents in the control group, even when controlling for crystallized intelligence. Difficulties in working memory and attention appear to be key cognitive features in children and adolescents with Noonan syndromes and should be taken into account when problems in daily life occur (eg, learning problems). Although we found no relationship between executive functions and social cognition using neuropsychological tasks, social functioning in daily life may very well be negatively affected by working memory and attention problems. When such basic information processes do not run smoothly, it is difficult to function well in a complex social context. Therefore, clinicians should consider difficulties in working memory and attention when interpreting problems in social functioning. Interventions to improve social functioning should also pay attention to these difficulties in executive functioning, besides targeting emotion regulation and social skills.

Future studies could focus on improving executive functioning or compensating for difficulties in executive functioning in children and adolescents with Noonan syndromes to investigate whether this leads to an improvement in daily functioning. To enhance social functioning in daily life, the development of an interactive social–emotional training program targeting emotion regulation and social skills in children and adolescents with Noonan syndromes would be highly beneficial. Currently, the first steps toward the development of a social–emotional training program for young people with Noonan syndromes are taken, based on the social–emotional training program for adults with NS.^42^ This training program incorporates psychoeducation, strategies, and exercises to improve emotion recognition (in oneself and others), social perception, and mentalization, and the verbalization and regulation of emotions. To conclude, this study illustrates that individual neuropsychological assessment is important in children and adolescents with Noonan syndromes, and should always be supplemented by behavioral measures to provide a complete picture of individual strengths and weaknesses.
