# Supplementary material for: Executive and Social Functioning in Children and Adolescents With Noonan Syndromes: Cognition and Behavior
Source: JAACAP Open. 2025 May 21;3(3):645–54. doi: 10.1016/j.jaacop.2025.05.002 (PMC12414317; doi:10.1016/j.jaacop.2025.05.002)
Supplement: Supplemental Material [file mmc1.docx]

Supplement 1

Cognitive measures

*Intelligence*

To assess crystallized intelligence, the Verbal Comprehension Index (VCI) of the Wechsler Intelligence Scale for Children, fifth edition (WISC-V-NL^1^) was administered. The VCI, consisting of the two subtests Similarities and Vocabulary, was used as a measure of verbal comprehension. According to Egberink and de Leng^2^, the WISC-V-NL displays good reliability and adequate construct validity, but inadequate criterion validity due to a lack of research. Higher scores indicate higher abilities in the area of verbal reasoning, but also higher crystallized intelligence. Crystallized intelligence is defined as a person’s acquired language-based knowledge against the background of her or his culture and environment^3^ and is therefore a good representation of general intellectual capacities without a strong reliance on executive functioning.

*Working memory*

The Digit Span subtest of the WISC-V-NL^1^ was used to assess working memory. Here, participants had to complete three conditions (i.e., repeating numbers forwards, repeating numbers backwards, and sorting and repeating numbers in ascending order). In all conditions the number of digits increase as the task progresses. For each correctly completed sequence, participants receive one point. The total score was calculated by summing up the scores of the three conditions, with higher scores indicating higher working memory capacities.

*Inhibitory control*

The Color Word Interference Test (CWIT) of the Delis-Kaplan Executive Function System (D-KEFS^4^) was used to assess inhibitory control. In condition 1, children and adolescents were asked to name the ink color (red, green or blue) of presented squares. In condition 2, they had to read the names of the color words ‘red’, ‘green’, and ‘blue’. In condition 3, they were asked to name the ink colors of incongruent color words (e.g., the word ‘green’ printed in blue ink; correct answer is ‘blue’). In condition 4, they had to switch between the second and third condition. When a color word was printed in a rectangle, participants were asked to read the names of the color words. When the color word was not printed in a rectangle, participants were asked to name the ink colors of incongruent color words. The time it took participants to complete the task was recorded for all conditions. A ratio score was calculated by dividing the time on condition 3 (i.e., naming colors of incongruent printed color words) by the time on condition 1 (i.e., naming colors), which was used as a measure of inhibition. The test-retest reliability (*α* *=* .77-.90) and internal consistency (*α =* .72-.86) were rated as good.^4^ Higher ratio scores indicate lower inhibitory control.

*Cognitive flexibility*
The Trail Making Test (TMT*)* of the D-KEFS^4^ was used to assess cognitive flexibility. It consists of five conditions: visual scanning, number sequencing, letter sequencing, number-letter switching, and motor speed. In condition 1, participants were asked to cross out a specific number, in condition 2, to connect numbers in ascending order and in condition 3, to connect letters in alphabetical order. In condition 4, they were asked to connect numbers and letters in ascending or alphabetical order respectively, while alternating between letters and numbers. In the last condition, they had to follow a dotted line as quickly as possible, connecting circles on the page. The time required to complete each task was recorded for all conditions. A ratio score was calculated by dividing the time on condition 4 (i.e., number-letter switching) by the average time of conditions 2 (i.e., number sequencing) and 3 (i.e., letter sequencing), which was used as a measure of cognitive flexibility. The internal consistency (α = .60-.81) and test-retest reliability (α = .80) were rated as average to good.^4^ Higher ratio scores indicate lower cognitive flexibility.

*Sustained attention*Concentration performance of the D2 Test of Attention^5^ was included to assess sustained attention. In this cancellation task, participants were asked to cross out target symbols (i.e., the letter ‘d’ with exactly two dashes) and to ignore other symbols that serve as distraction. Participants had to cross out as many characters as possible within 20 seconds per row and then move on to the next row. The d2 consists of a total of 14 rows. Concentration performance was calculated by subtracting the total number of incorrectly crossed targets from the total number of correctly crossed targets.^6^ According to Egberink and de Leng^7^, the D2 Test of Attention displays insufficient reliability, construct validity and criterion validity due to a lack of research. Higher scores indicate better sustained attention.

*Emotion recognition*

The subtest Affect Recognition of the Developmental Neuropsychological Assessment (NEPSY-II-NL^8^) was administered to assess emotion recognition and consists of four subtasks. Participants were shown photographs of children’s faces with different emotional expressions. The emotions fear, anger, sadness, happiness, and disgust were expressed in different intensities, complemented by neutral facial expressions. First, participants were asked to indicate whether two faces showed the same emotional expression or not. Second, participants were asked to select the photographs with the same facial expression. Third, participants had to select photographs that showed the same emotional expression as a photograph at the top of the page. Finally, participants had to remember the emotion of the facial expression shown and select photographs with the same facial expression. Total scores were obtained by summing up the correct answers on the four subtasks. According to Egberink and de Leng^9^, the NEPSY-II demonstrates insufficient reliability, construct validity and criterion validity due to a lack of research and unclear results. Higher scores indicate higher abilities in emotion recognition.

*Theory of Mind*

The Theory of Mind subtask of the NEPSY-II-NL^8^ was used to assess mentalizing abilities. It consists of verbal tasks and contextual tasks that assess the ability to understand mental functions, to understand that others have their own thoughts, ideas and feelings and to understand how emotions are related to social context. The verbal tasks consist of different scenarios that were read or shown to participants. Participants were asked to answer questions related to another individual's point of view. In the contextual tasks, pictures of social situations were shown to participants and they were asked to select pictures of the most appropriate emotional facial expression in this social situation. Total scores were calculated by summing up the correct answers of the verbal and contextual tasks, with higher scores indicating better Theory of Mind, or in other words mentalizing skills.

Behavioral measures

*Executive functioning*

The Behavior Rating Inventory of Executive Function (BRIEF^10^) was used to assess everyday EF problems experienced in the home environment of children/adolescents reported by parents. In this questionnaire, parents of the participating children/adolescents were asked to rate the likelihood of their children exhibiting specific behaviors in the past six months. The parents had to rate these behaviors (e.g., ‘*can concentrate only briefly*’ on a three-point Likert scale from 1 (never) to 3 (often). The BRIEF consists of 75 items that result in eight subscales (i.e., inhibition, shifting, emotional control, initiation, working memory, planning and organization, organization of materials, and monitor). The Dutch version of the BRIEF has a high test-retest reliability and a very high internal consistency.^11^ According to Egberink and de Leng^12^, the BRIEF shows adequate reliability, but insufficient construct and criterion validity due to a lack of research. In our sample a high internal consistency was found (Cronbach’s α = .98). In this study, total scores were used, which were calculated by summing up the scores on the eight subscales. Total scores ranged from 72 to 216, with higher scores indicating more EF problems experienced in daily life.

*ADHD characteristics*

The ADHD-questionnaire (AVL^13^) was used to assess ADHD characteristics. The AVL was completed by parents of the participants and assessed the extent to which the child/adolescent has exhibited behaviors that are associated with ADHD over the last six months. On a four-point Likert scale from 0 (not at all or barely) to 4 (very often (daily)), child/adolescent behaviors were rated, for example the behavior ‘Moves restlessly with hands and feet, fidgets, wiggles back and forth’. The AVL consists of 18 items that can be divided into three subscales, namely ‘attention-deficit’, ‘hyperactivity’ and ‘impulsivity’. The internal consistency (Cronbach’s α = .96), inter-rater reliability (α = .88) and test-retest reliability (α = .95) of the questionnaire were found to be high.^13^ As stated by Egberink and de Leng^14^, the AVL demonstrates good reliability, good construct validity and adequate criterion validity. A high internal consistency was found in our sample (Cronbach’s α = .94). The total score was calculated by summing up all items, with higher scores indicating that children/adolescents show more ADHD characteristics in daily life.

*ASD characteristics*

The Autism spectrum questionnaire (ASV^15^) was used to assess ASD characteristics. In this questionnaire, parents of participating children/adolescents were asked to rate 24 items that can be divided into the two subscales ‘interactive and communication skills’ and ‘strange, deviant behavior patterns’. On a five-point Likert scale from 1 (completely disagree) to 5 (completely agree), child/adolescent behaviors were rated, for example the behavior ‘Takes own and others' interests into account’. According to Egberink and Leng^16^, the ASV displays good reliability, adequate construct validity and adequate criterion validity. In our sample a high internal consistency was found (Cronbach’s α = .95). The total score was calculated by summing up all items, with higher scores indicating more total behavioral characteristics of ASD in daily life.

Table S1

Pearson Correlations Between all Variables

|  | 1 | 2 | 3 | 4 | 5 | 6 | 7 | 8 | 9 | 10 |
| --- | --- | --- | --- | --- | --- | --- | --- | --- | --- | --- |
| 1. Age | - |  |  |  |  |  |  |  |  |  |
| 2. Crystallized intelligence | -.087 | - |  |  |  |  |  |  |  |  |
| 3. Sustained attention | .670*** | .244 | - |  |  |  |  |  |  |  |
| 4. Working memory | .416** | .338* | .632*** | - |  |  |  |  |  |  |
| 5. Inhibitory control | -.108 | -.379** | -.409** | -.152 | - |  |  |  |  |  |
| 6. Cognitive flexibility | -.072 | -.167 | -.057 | -.124 | .117 | - |  |  |  |  |
| 7. Emotion recognition | .163 | .389** | .338* | .270 | -.211 | -.180 | - |  |  |  |
| 8. Theory of Mind | .416** | .345* | .406** | .255 | -.270 | -.368** | .404** | - |  |  |
| 9. EF problems | .180 | -.489*** | -.212 | -.335* | .212 | -.235 | -.234 | .148 | - |  |
| 10. ADHD characteristics | .075 | -.339* | -.301* | -.353* | .138 | -.272 | -.166 | .166 | .934*** | - |
| 11. ASD characteristics | .143 | -.378** | -.215 | -.329* | .196 | -.236 | -.213 | .072 | .873*** | .786*** |

*Note:* *p < .05, **p < .01, ***p <.001, ADHD = attention-deficit/hyperactivity disorder, ASD = autism spectrum disorder, EF = executive function.

**Supplemental references**

¹Wechsler D. WISC-V-NL. Wechsler Intelligence Scale for Children – Fifth Edition – Nederlandstalige bewerking: Afname- en scoringshandleiding [WISC-V Dutch version: Administration and Scoring Manual]. Pearson; 2018.

²Egberink IJL, de Leng WE. COTAN beoordeling, WISC-V-NL [COTAN review, WISC-V-NL]. Boom uitgevers Amsterdam; 2019. Accessed December 6, 2024. https://cotandocumentatie.nl

³Weiss LG, Holdnack JA, Saklofske DH, Prifitera A. Theoretical and clinical foundations of the WISC-V index scores. In: Weiss LG, Saklofske DH, Holdnack JA, Prifitera A, eds. WISC-V Assessment and interpretation: Scientist-practitioner perspective. Elsevier; 2016:97-121.

⁴Delis DC, Kaplan E, Kramer J. Delis-Kaplan Executive Function System. Psychological

Corporation; 2001.

⁵Kraan M. D2 Aandachts- en concentratietest: Handleiding [Attention- and concentration test: Manual]. Hogrefe Uitgevers B.V.; 2007.

⁶Bates ME, Lemay EP. The d2 Test of Attention: Construct validity and extensions in scoring techniques. J Int Neuropsychol Soc. 2004;10(3):392-400. https://doi.org/10.1017/S135561770410307X

⁷Egberink IJL, de Leng WE. COTAN beoordeling, d2 [COTAN review, d2]. Boom uitgevers Amsterdam; 2008. Accessed December 6, 2024. https://cotandocumentatie.nl

⁸Zijlstra HP, Kingma A, Swaab H, Brouwer WH. NEPSY-II-NL Nederlandstalige bewerking: Afnamehandleiding [NEPSY-II-NL Dutch version: Administration manual]. Pearson Assessment and Information B.V.; 2010.

⁹Egberink IJL, de Leng WE. COTAN beoordeling, NEPSY-II-NL [COTAN review, NEPSY-II-NL]. Boom uitgevers Amsterdam; 2011. Accessed December 6, 2024. https://cotandocumentatie.nl

¹⁰Huizinga M, Smidts DP.  BRIEF Vragenlijst executieve functies voor 5 tot 18-jarigen [BRIEF Questionnaire executive functions for 5- to 18-year-olds]. Hogrefe Uitgevers B.V.; 2012.

¹¹Huizinga M, Smidts DP. Age-related changes in executive function: A normative study with the Dutch version of the Behavior Rating Inventory of Executive Function (BRIEF). Child Neuropsychol. 2010;17(1): 51-66. https://doi.org/10.1080/09297049.2010.509715

¹²Egberink IJL, de Leng WE. COTAN beoordeling, BRIEF [COTAN review, BRIEF]. Boom uitgevers Amsterdam; 2014. Accessed December 6, 2024. https://cotandocumentatie.nl

¹³Scholte EM, Van der Ploeg JD. ADHD-vragenlijst [ADHD-questionnaire]. Swets & Zeitlinger; 1998.

¹⁴Egberink IJL, de Leng WE. COTAN beoordeling, ADHD-vragenlijst [COTAN review, ADHD-questionnaire]. Boom uitgevers Amsterdam; 2005. Accessed December 6, 2024. https://cotandocumentatie.nl

¹⁵Van der Ploeg JD, Scholte EM. Autisme Spectrum Vragenlijst (ASV) Handleiding [Autism spectrum questionnaire manual]. Bohn Stafleu van Loghum; 2014.

¹⁶ Egberink IJL, de Leng WE. COTAN beoordeling, Autisme Spectrum Vragenlijst [COTAN review, Autism spectrum questionnaire]. Boom uitgevers Amsterdam; 2015. Accessed December 6, 2024. https://cotanldocumentatie.nl
